# Death of Dr. Wilson, and Resolutions Runnels County Medical Society

**Published:** 1887-09

**Authors:** 


DIED. Dr. W. H. Wilson, at Runnels, Texas, on August the 4th, 1887, of cancer of the stomach.

RESOLUTIONS BY THE RUNNELS COUNTY MEDICAL ASSOCIATION ON THE DEATH OF W. H. WILSON, M. D.

WHEREAS, it has pleased our Heavenly Father in the exercise of his most exalted providence to remove from his respective sphere of usefulness, and from his many companions in the professional brotherhood, the late W. H. Wilson, M. D., and,

WHEREAS, we realize with pathetic feeling the loss we have thus sustained, we deem it our melancholy duty to give expression to the irreparable vacuum in our midst, and the appreciation of his worth as a physician and friend; therefore be it

*Resolved*, That by the death of Dr. Wilson, the medical profession of Runnels county has been bereft of one of its most efficient and faithful members.

*Resolved*, That the Runnels County Medical Association has been deprived of one of its most earnest supporters and co-workers, and whose thoughtful counsel placed him in the front ranks of its membership.

*Resolved*, That we as individuals are painfully sensitive to our loss. As a confere he was ethical and non-egotistically observant of the feelings and interests of his fellowman. As a man, both brave and truthful, honest and just. Endowed with a positive character, he departed this life.

Dr. Wilson had many loving friends to appreciate him; enemies also had he, but even they respected him.

*Resolved*, That we tender the bereaved family of deceased our heartfelt sympathy as professional brothers, and invoke that consolation for them which can only be given by Him who directeth all things.

*Resolved*, That these resolutions shall be placed upon the Minutes of Runnels County Medical Association. That the family of deceased be furnished with a copy and DANIEL'S MEDICAL JOURNAL be asked to publish the same.

